# Off the Market: The Percentage of Products Available After Ten Years

**DOI:** 10.5435/JAAOSGlobal-D-17-00076

**Published:** 2018-01-03

**Authors:** Eric L. Smith, Jonas Miller, Taryn LeRoy, Eitan Ingall, David J. Tybor, Joseph T. Martin, Mary E. Pevear

**Affiliations:** From the Division of Orthopaedic Surgery, Boston Medical Center, Boston, MA (Dr. Smith and Ms. Pevear); Louisiana State University School of Medicine, New Orleans, LA (Mr. Miller); Tufts University School of Medicine, Boston, MA (Dr. LeRoy and Dr. Tybor); Boston University School of Medicine, Boston, MA (Dr. Ingall); and Vanderbilt University College of Arts and Sciences, Nashville, TN (Mr. Martin).

## Abstract

**Background:**

We observed that medical devices advertised in journals are often no longer available 5 to 10 years after first being advertised. In this study, we quantified the percentage of products advertised from 2003 to 2008 in the *Journal of Bone and Joint Surgery, American,* which were still available 5 to 10 years after first being advertised.

**Methods:**

We created a database of 427 unique orthopaedic products advertised in the *Journal of Bone and Joint Surgery*. In 2013, we classified products into categories: available in advertised form, available in modified form, available under a different manufacturer, and available but temporarily recalled, discontinued voluntarily, or discontinued by forced recall.

**Results:**

A total of 13.8% of products were discontinued 5 to 10 years after being advertised. Three percent were discontinued through forced recall, and 10.8% were discontinued voluntarily. Of the products still available, 60.2% were in current form, 12.9% were modified, 11.9% were available under a different company, and 1.2% were available but were temporarily recalled.

**Conclusion:**

Five to 10 years after the initial advertisement, nearly 40% of products were not available in their original advertised form.

Pharmaceutical companies and device manufacturers use well-respected journals with large readerships to reach their audience. Journal advertising is one of the most effective methods of promoting new medical products.^1,2^ Although advertising provides journals and publishing companies with significant revenue, there can be discord between the financial motivations of for-profit company advertisements and the scientific objectives of the peer-reviewed journal.^3^ Peer-reviewed articles go through an extensive review process, beginning with the editor-in-chief and deputy editor and then passed on to two or three reviewers to ensure high-quality literature. However, when it comes to advertisements, no such guidelines exist. Despite the reputation of these journals, the quality of products advertised may not be equivalent to the level of evidence of peer-reviewed articles. Orthopaedic surgeons must differentiate between product promotion and clinical merit.^4^

Surgeons at an academic medical center observed a trend in which medical devices advertised in peer-reviewed journals were no longer available 5 to 10 years after being advertised. Further investigation of the trend determined that only two previous assessments of the validity of claims made in print advertising have been done^3,5^ in the orthopaedic surgery literature. Both studies found that approximately half of the claims made were supported by the literature, suggesting that orthopaedic surgeons may not have adequate evidence to make educated decisions regarding their medical device choices by reading the advertisement alone.

The purpose of this study was to evaluate the availability of products advertised in a high-impact orthopaedic journal 5 to 10 years after observing the advertisement. We hypothesized that a substantial portion of these advertised products would have become discontinued or modified within this time frame.

## Methods

### Selection of Advertisements

The time period from 2003 to 2008 allowed us to achieve between 5 and 10 years of follow-up of the products. To attain a sample of widely distributed orthopaedic products, we selected a top-tier orthopaedic journal, the *Journal of Bone and Joint Surgery, American,* and included all volumes published between January 2003 and December 2008. We manually reviewed each paper journal issue and cataloged all advertisements of orthopaedic products. We recorded the device name, model, and manufacturer. After separating advertisements based on the product type, we consulted with orthopaedic surgeons from various specialties to establish functional categories for analysis: implantable devices, medications, surgical tools/accessories, or biological products. Each product was given one categorization. Advertisements for companies, conferences, and continuing education opportunities were excluded. Our cataloged sample included a total of 1,562 advertisements. All relevant product information was transcribed into a Microsoft Excel spreadsheet, including advertisement date, manufacturer, make, model, and surgical function. Using this preliminary spreadsheet, we designed a database by grouping products by company, make, and model. For each unique product, date of advertisement publication and total number of ads featuring the product were added. Duplicate products were counted as single entries. Modified and remodeled products were maintained as separate entries. Our final database consisted of 427 unique advertised products.

### Follow-up Product Status Classification

The follow-up period varied for each product according to the initial advertisement date and the date of discontinuation or modification. All products were reviewed in 2013, yielding a product follow-up time of 5 to 10 years from the initial advertisement date. Sixteen manufacturers comprised over 61% of our sample.

During this 2013 retrospective review of product status, we used a multifaceted approach to obtain product information. We researched each product extensively using industry press releases and company websites to determine whether it was still on the market under the same design. Any recalled or modified product was typically documented in company press releases. In some instances, smaller companies or branches were sold to larger device manufacturers, in which case it was documented. If a product's status was not documented on a company's website, we then consulted with medical device databases published by the federal government, including the FDA's Manufacturer and User Facility Device Experience, Medical Device Reporting, and Recalls of Medical Device databases. In addition, we used industry-standard databases such as DynaMed to research the current availability of advertised medications.

We then documented product status based on the following classifications: available (current form), available (modified), available (under a different company), available (temporary recall), discontinued (voluntarily), and discontinued (forced recall). The research methods described earlier were used to determine product classification. Final product status classification (Table 1) was made by unanimous decision, and data were entered into the database for analysis.

**Table 1:**
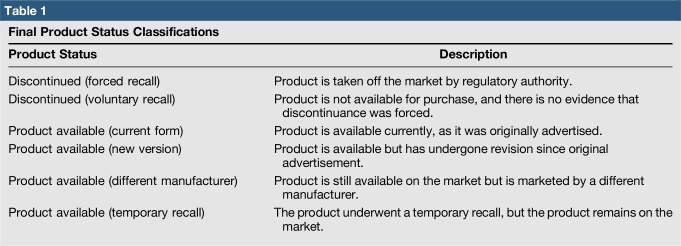
Final Product Status Classifications

## Results

**Figure 1:**
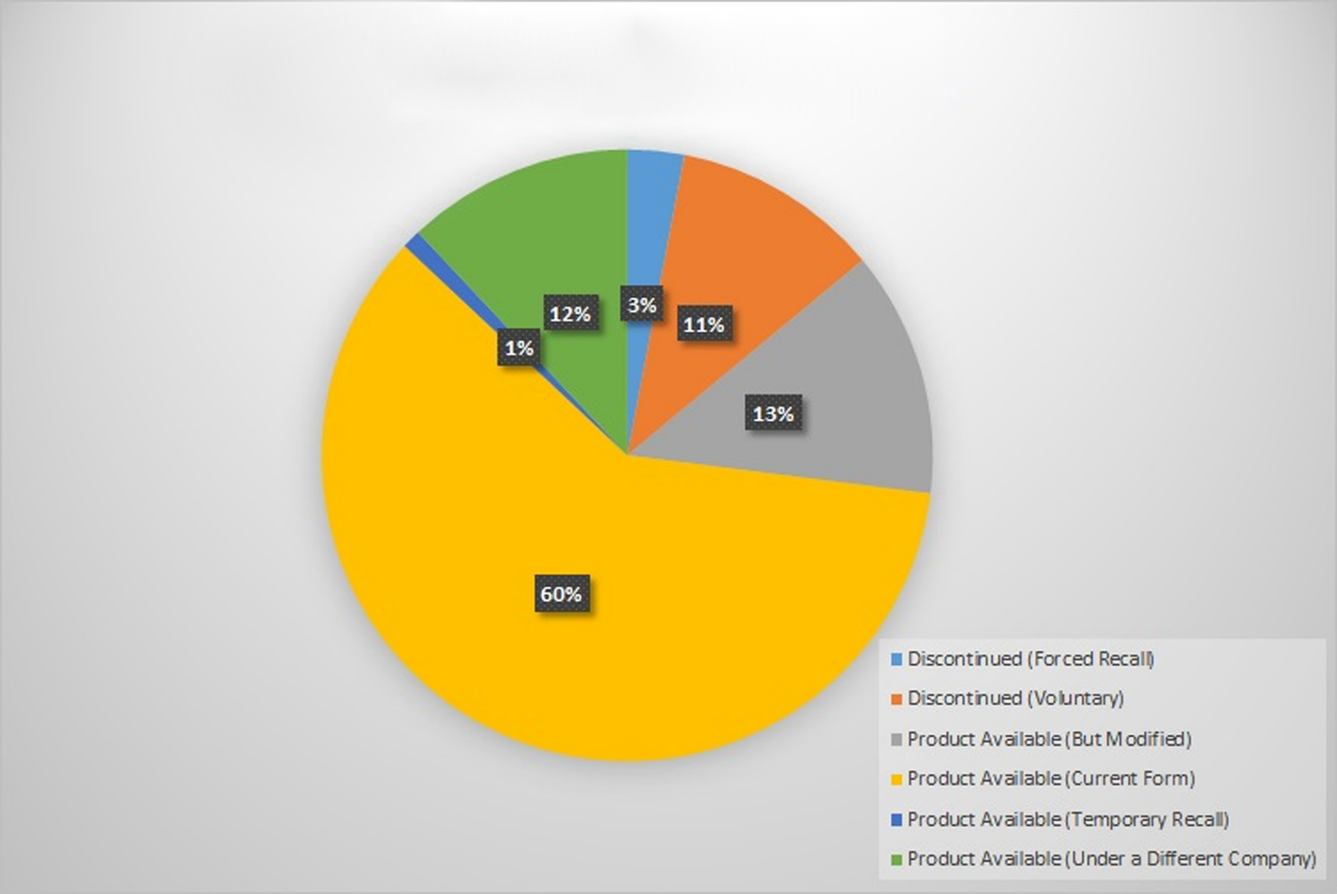
Pie chart illustrating the breakdown of product status 5 to 10 years after being initially advertised.

Of the 427 medical products assessed, we found that 13.8% were deemed discontinued according to the above classification scheme 5 to 10 years after being advertised (Figure 1). Three percent were discontinued by forced recall, and 10.8% were discontinued voluntarily. Most notable were total hip arthroplasty products, which had a discontinuance rate of 19%. Of the products available, 60.2% were in current form, 12.9% were modified, 11.9% were available under a different company, and 1.2% were available but had been temporarily recalled at some point during the product follow-up period.

**Figure 2:**
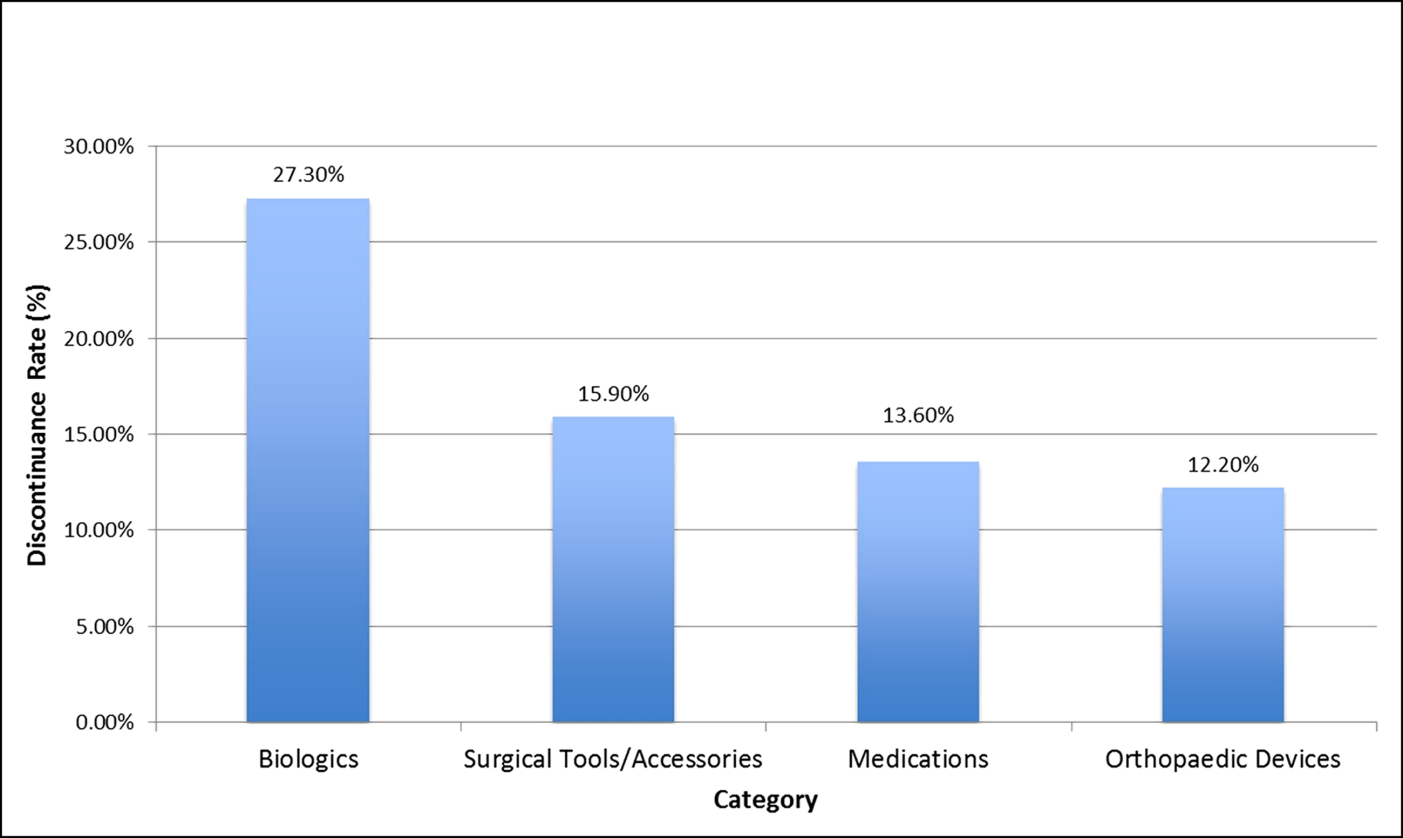
Bar graph showing discontinuance rates broken down further by product category.

An analysis of the discontinuance rate based on the product subcategory was also done (Figure 2). Biologics had the highest discontinuance rate (27.3%), followed by surgical tools/accessories (15.9%), medications (13.6%), and orthopaedic implantable devices (12.2%).

## Discussion

Orthopaedic surgeons trust peer-reviewed journals as sources of the high-quality, evidence-based literature. Advertisements in peer-reviewed journals, therefore, may have a considerable influence on the medical products and innovative technologies surgeons choose to adopt.^6^ Medical devices play an ever-increasing role in our healthcare system, as profit-seeking companies develop newer and more expensive implants to improve patient outcomes. According to the FDA, the medical device industry has experienced tremendous growth in both revenue and the technical complexity of products; however, serious adverse events related to medical devices have outpaced industry growth, and gaps in medical device quality can pose a significant risk to patients.^7^ In a field that strives for developing innovative technology, there exists a cause for concern when high-profile and highly advertised devices, most notably metal-on-metal bearing arthroplasty, are removed from the market because of device malfunction.^2^

Estimated to be the most profitable strategy for medical device manufacturers, advertising is at the core of the medical device industry.^6,8^ Print advertising in journals allows for higher message penetration. In the pharmaceutical industry, physician exposure to advertisements has been linked to an increase in the prescription of advertised drugs.^9^ Print sources, including peer-reviewed journals, influence the clinical practices orthopaedic surgeons use and are a forum for orthopaedic surgeons to gather information on which products to use.

However, to date, there is no regulation of the content of these advertisements, leading to questions about the validity of claims made by product advertisements. Of course, criticisms of these claims go well beyond the scope of the orthopaedic surgery literature. A multitude of studies have criticized the pharmaceutical industry's direct-to-physician advertising in medical journals. Wilkes et al^12^ looked at 109 pharmaceutical advertisements and surveyed physicians about the validity of the claims made in the advertisement. In 44% of the advertisements, reviewers felt that the content could lead to improper drug prescribing. Reviewers would not have recommended the publication of 28% of advertisements. Yet another study looking across four general medical journals and a 6-month sample of advertisements noted that only 33 of 187 distinctive advertisements contained quantitative results.^10^ In another study looking at advertisements in a prominent otolaryngology journal, only 14 of 50 (28%) claims were supported based on strong evidence.^11^

The validity of advertisement claims has also been evaluated in the orthopaedic surgery literature. A study by Bhattacharya et al^5^ evaluated claims made in print advertisements across several prominent orthopaedic journals. These authors contacted the companies that published the advertisements and requested to evaluate the data on which the advertisement claim was based. For 52% of the claims, all three surgeon reviewers agreed that the data were not strong enough to be used in clinical practice. By contrast, all three reviewers agreed that only 14% of claims were appropriately well supported by the data. Notably, only 24% of these advertisements provided a citation for the stated claim. In a similarly conducted review, Davidson and colleagues found that 76% of claims were considered by two or more reviewers to be “weak” and 36% (18/50) claims referenced no supporting evidence.^6^

In our study, we evaluated the survivorship of orthopaedic products advertised in a first-tier orthopaedic journal. Overall, results from our study indicated that at 5 to 10 years after the initial advertising, nearly 40% of products were not available in their original advertised form. The device discontinuance rate of orthopaedic products was 13.8%. Three percent were discontinued by forced recall, and 10.8% were discontinued voluntarily. Our study did not assign a cause of discontinuance.

Product survivorship as a surrogate for longer term product value and clinical efficacy has not been rigorously studied but is indeed a logical proxy. Although we recognize that there is a natural evolution of industry with products entering and exiting the market, surgeons must be aware that a device may be unavailable as advertised several years later. This should give a pause to the journal reader and be the impetus for rigorous product investigation before clinical implementation. The *Journal of Bone and Joint Surgery* America is one of the most valued sources of information for orthopaedic surgeons and researchers and is considered a top-tier journal because of its peer-reviewed, well-trusted scientific process. Despite this, of the 427 industry products we assessed, only 60.2% exist 5 to 10 years later in their original form. All articles published in the journal are peer reviewed, but based on our findings, advertisements do not necessarily undergo the same level of review. To this end, the results of our study suggest the need for a greater degree of high-quality evidence for products and a higher level of peer review for advertisements before they appear in print.

This study does have limitations. It is possible that some products entered into our database could have remained in their current state with their names changed. We are unaware of any data pertaining to the rate of device name change without functional or design change in the orthopaedic industry. When searching for product status during our review, we broadly searched for products by both name and function with the intent of identifying these name changes. We did not identify any products that had sole changes in product name without some change in function or design. Second, given that there is no single repository for all orthopaedic products on the market, it is possible that our search was not all encompassing, thus potentially missing certain products and incorrectly assigning their follow-up status. We assumed that most products would be listed on the manufacturer's website and did indeed find most products in this manner. In the event that we did not locate the product, we undertook an exhaustive search of all known databases. Another limitation of this study is that we are evaluating only one journal, albeit a highly regarded one, of a large number of orthopaedic journals. Whether these results can be generalized to advertisements in other orthopaedic journals or other disciplines will require further rigorous investigation. Finally, as noted earlier, we were unable to determine the natural course of device discontinuation in the orthopaedic product market at large. Indeed, our findings could simply represent “normal product shelf life” with respect to the pace of technological advancement and obsolescence. If our results do simply capture this so-called product natural history, we maintain that it is important for practicing surgeons to be mindful of this as they choose the products to incorporate into their practice.

We chose the reference years 2003 to 2008 to achieve a minimum of 5-year follow-up to the advertised product, as we felt that this was a clinically relevant follow-up period. This snapshot in time of the advertised product does not represent the first or last point at which the products were advertised but rather reflects the marketing strategies of the company manufacturing the product. In other words, someone within the company felt that the product warranted the purchase of an advertisement to increase sales. Our research could have sampled advertised products that were undergoing a natural evolution in improvement. However, given the fact that advertisements cost several thousands of dollars, the company likely felt strongly enough at that time to market the product irrespective of what the next generation of product would be.

More specifically, for example, the ability of a company to advertise the latest highly cross-linked polyethylene, then advertise the next month a new-and-improved highly cross-linked polyethylene, leaves the surgeon wondering if there was something wrong with the originally marketed polyethylene.

In conclusion, only 60.2% of products advertised were available 5 to 10 years after initially being advertised in the *Journal of Bone and Joint Surgery*. This number is informative, given the well-documented history of unsupported claims made in advertisements appearing in medical journals, including in the orthopaedic surgery literature. We urge orthopaedic surgeons to practice caution while choosing a novel product based on print advertisements to use in their practice and advise them to gather more information about the product before its implementation in a clinical setting.
